# Smoking, second-hand smoke exposure and smoking cessation in relation to leukocyte telomere length and mortality

**DOI:** 10.18632/oncotarget.11051

**Published:** 2016-08-04

**Authors:** Wahyu Wulaningsih, Fidel Emmanuel C. Serrano, Adi Utarini, Tetsuya Matsuguchi, Johnathan Watkins, for PILAR Research Network

**Affiliations:** ^1^ Division of Cancer Studies, King's College London, London, UK; ^2^ MRC Unit for Lifelong Health and Ageing, University College London, London, UK; ^3^ Division of Haematology/Oncology, Faculty of Medicine, Universitas Gadjah Mada, Yogyakarta, Indonesia; ^4^ PILAR Research and Education, Cambridge, UK; ^5^ National Institute of Molecular Biology and Biotechnology, University of The Philippines Diliman, Diliman, Quezon City, Metro Manila, The Philippines; ^6^ Department of Health Policy and Management, Faculty of Medicine, Universitas Gadjah Mada, Yogyakarta, Indonesia; ^7^ Department of Biochemistry and Biophysics, University of California, San Francisco, CA, USA; ^8^ Driver Group, L.L.C., San Francisco, CA, USA; ^9^ Institute for Mathematical and Molecular Biomedicine, King's College London, London, UK

**Keywords:** smoking, second-hand smoke exposure, smoking cessation, telomere, ageing

## Abstract

**Objectives:**

To investigate the link between smoking exposure, telomere length and mortality, with emphasis on second-hand smoke (SHS) exposure and the duration of smoking cessation.

**Results:**

A total of 1,018 participants died during follow-up (mean: 10.3 years). A 50 base-pair decrease in LTL was shown among cotinine-confirmed current versus never smokers. The 90^th^ quantile of LTL decreased with increasing cotinine among never smokers, indicating a role of SHS. Longer telomeres with smoking cessation were indicated but limited to a 3-16 year period of abstaining smoking. When assessing mortality, we observed a lower risk of all-cause death for the second quintile compared to the first among never smokers (HR: 0.67, 95% CI: 0.52-0.87), and a higher risk was found among current smokers (HR: 1.89, 1.19-2.92).

**MATERIALS AND METHODS:**

We studied 6,456 nationally representative U.S. respondents with mortality follow-up through to 31 December 2011. Smoking status was assessed by interviews and cotinine levels. Relative leukocyte telomere length (LTL) was quantified by polymerase chain reaction (PCR). Multivariable linear regression was performed to examine LTL by smoking exposure, adjusted for age, sex, race/ethnicity, socioeconomic status, education, body mass index, alcohol consumption, and physical activity. We further estimated the association of LTL with cotinine levels using quantile regression, and with smoking cessation dynamics. Cox regression was used to estimate mortality by smoking status and LTL.

**Conclusion:**

Our findings indicated a complex association between smoking, telomere length, and mortality. LTL alterations with SHS and smoking cessation warrant further investigation for translation to public health measures.

## INTRODUCTION

Tobacco use is one of the leading global risk factors for mortality, particularly from chronic diseases including heart disease, diabetes and cancer [1, 2]. Cigarette smoking alone is considered responsible for up to 71% of lung cancer deaths worldwide [3]. With the causal relationship between smoking and lung cancer well established, [4] recent studies have further focused on mitigating the risk for never smokers as well as former smokers. To this end, understanding the extent of how second-hand smoke (SHS) exposure and smoking cessation affect smoking-related health outcomes is important to inform public policies.

Because a wide variety of diseases associated with smoking may be classified as age-related, ageing has emerged as in important comparative measure of health outcomes with regards to smoking and SHS exposure. Telomeres are nucleoprotein complexes that localize at the ends of eukaryotic chromosomes and perform functions for chromosomal protection, integration and replication [5]. Due to the end-replication problem, each somatic mitotic division results in the loss of telomeric repeats by 30 to 200 base pairs, leading to gradual telomere shortening [6]. Thus, telomere length can be used as a standard biomarker for cellular aging. Chronic exposure to biological insults such as oxidative stress may result in significantly shortened telomeres which in turn leads to cellular senescence and apoptosis [7, 8].

As a source of free radicals that may elicit oxidative stress and inflammation, [9] smoking has been hypothesized to promote telomere shortening, accelerating the ageing process and increasing the chances of developing age-related diseases [10, 11]. An inverse correlation between active smoking and telomere length has been indicated in some studies, [12–17] but little is known about their joint impact on mortality. Moreover, associations between SHS or smoking cessation and telomere length in the general population are rarely discussed. Therefore, to gain further insight into the association between smoking and longevity, we aimed to characterise associations between smoking exposures in relation to telomere length and mortality in a representative sample of the U.S. population.

## RESULTS

Characteristics of study participants and corresponding mean telomere length are shown in Table 1. Mean age of study participants was 45.9 years. A total of 1,018 (16%) persons died during follow-up (mean: 10.3 years). Nearly half of the population (49%) were never smokers. Around 15% of never smokers reported SHS exposure either at home or at workplace. Mean LTL decreased with age (Supplementary Table S1, Supplementary Data), and was found to be longer among SHS-exposed as compared to SHS-unexposed never smokers (Supplementary Figure S1, Supplemental Information).

**Table 1:** Weighted characteristics of study participants and mean telomere length

	N	Weighted %	Mean LTL in kbp (SE)
**Age, years**			
20–30	1028	17.64	6.17 (0.04)
30–40	1112	21.23	5.97 (0.04)
40–50	1215	22.82	5.87 (0.04)
50–65	1472	21.89	5.65 (0.04)
≥65	1629	16.40	5.42 (0.04)
**Sex**			
Male	3333	49.99	5.83 (0.04)
Female	3123	51.01	5.81 (0.03)
**Race/ethnicity**			
Non Hispanic white	3337	74.46	5.79 (0.04)
Non Hispanic black	1108	9.06	5.96 (0.05)
Mexican American	1540	6.83	5.82 (0.05)
Other	471	9.65	5.91 (0.07)
**PIR**			
<1	1115	13.82	5.90 (0.06)
1–2	1656	20.50	5.77 (0.05)
2–3	1051	15.63	5.80 (0.04)
≥3	2634	50.06	5.83 (0.04)
**Education**			
Less than high school	2129	20.73	5.71 (0.04)
High school	1510	25.87	5.81 (0.04)
Higher education	2817	53.40	5.87 (0.03)
**Cancer diagnosis**			
No	5911	92.04	5.84 (0.03)
Yes	545	7.96	5.61 (0.04)
**History of COPD**			
No	5992	92.12	5.84 (0.03)
Yes	464	7.88	5.63 (0.04)
**Vigorously active**			
No	5383	80.13	5.79 (0.03)
Yes	1073	19.87	5.96 (0.04)
**Body mass index (BMI), kg/m^2^**			
<18.5	92	1.69	5.88 (0.06)
18.5–25	1954	32.94	5.91 (0.04)
25–30	2357	35.16	5.80 (0.04)
≥30	2053	30.21	5.76 (0.03)
**Alcohol consumption**			
Never	479	6.89	5.91 (0.07)
Up to once a week	4715	70.65	5.80 (0.04)
2–3 times per week	640	11.93	5.91 (0.04)
4 times per week or more	622	10.53	5.78 (0.04)
**Self-reported moking status**			
Current smokers	1452	24.71	5.85 (0.03)
Former smokers	1751	25.35	5.71 (0.03)
Never smokers	3253	49.94	5.86 (0.04)
**Serum cotinine (ng/mL)^1^**			
<0.5	2633	40.00	5.79 (0.03)
0.5–10	2010	31.12	5.86 (0.04)
≥10	1641	28.82	5.84 (0.04)

1 Measured in 6,284 participants.

2 Second-hand smoke (SHS) exposure at home, defined as presence of one or more smokers at home for never and current smokers. For current smokers, exposure was defined as presence of two or more smokers at home.

3 Second-hand smoke (SHS) exposure at work, defined as being able to smell others' smoking at workplace.

When comparing telomere length across self-reported smoking status, we observed that being a current smoker was associated with a 50 bp decrease in LTL as compared to never smokers, with a 95% CI of −105 to 5 bp in the age- and race/ethnicity-adjusted model. Similarly weak associations were observed when comparing current to former smokers (−37, 95% CI: −87 to 12) or former to never smokers (−12, 95% CI: −51 to 27). Further adjustments did not alter these results (Supplementary Table S2, Supplementary Data). Although similar results were found in analysis using cotinine-confirmed smoking status, a 50 bp decrease (95% CI: −85 to −1) in LTL was shown among cotinine-confirmed current as compared to never smokers.

Among self-reported current smokers, increasing pack-year of cigarettes corresponded to shorter telomere length, with a 169 bp decrease in LTL (95% CI: −257 to −81) for 30 pack-year of cigarettes and over (P_trend_=0.002). Among former smokers, a 100 bp higher mean telomere length was observed in those who had ceased smoking for 10–20 years as compared to those who had ceased smoking for fewer than five years. Neither SHS exposure at home or at work or serum cotinine showed any associations with LTL (Table 2). Similar associations were seen when using cotinine-confirmed smoking status, and when excluding participants with cancer diagnosis or history of COPD (results not shown).

**Table 2:** Characteristics of smoking exposures and mean LTL by smoking status

	Weighted %	Mean LTL difference (bp)	95% CI
*Current smokers*			
**Years since started smoking**			
<5	6.38	Reference	Reference
5–10	12.55	−23	−233, 185
10–20	23.93	−47	−256, 161
≥20	57.14	48	−176, 273
P_trend_		0.61	
**No. of cigarettes smoked per day**			
<2	2.69	Reference	Reference
2–10	15.55	166	−93, 426
10–20	28.08	103	−126, 332
≥20	53.68	41	−176, 259
P_trend_		0.07	
**Pack-year of cigarette**			
<15	46.75	Reference	Reference
15–30	25.48	−76	−168, 14
≥30	27.77	−169	−257, −81
P_trend_		0.002	
**Log serum cotinine**		−20	−46, 5
*Former smokers*			
**Years since quitted smoking**			
<5	13.97	Reference	Reference
5–10	14.44	51	−60, 163
10–20	33.95	100	2, 197
≥20	37.64	113	−1, 227
P_trend_		0.05	
**Years smoking when quitting**			
<5	14.10	Reference	Reference
5–10	14.69	77	−80, 234
10–20	26.88	25	−103, 153
≥20	44.33	1	−107, 109
P_trend_		0.43	
**No. of cigarettes smoked per day when quitting**			
<2	8.23	Reference	Reference
2–10	22.86	−94	−220, 32
10–20	19.58	−105	−235, 25
≥20	49.32	−99	−239, 41
P_trend_		0.61	
**Pack-year of cigarette**			
<15	53.65	Reference	Reference
15-30	19.19	−63	−146, 19
≥30	27.16	−44	−148, 58
P_trend_		0.34	
**Log serum cotinine**		−7	−20, 6
*Never smokers*			
**SHS exposure at home^1^**	6.49	16	−109, 101
**SHS exposure at work^1^**	9.86	−8	−111, 95
**Log serum cotinine**		−8	−20, 4

All models were adjusted by age, sex, race/ethnicity, PIR, education, BMI, vigorous physical activity, and alcohol intake.

1 Second-hand smoke (SHS) exposure at home, defined as presence of one or more smokers at home for never smokers. Referents were those with negative responses to this definition.

2 Second-hand smoke (SHS) exposure at work, defined as being able to smell others' smoking at workplace. Referents were those with negative responses to this definition.

**Figure 1:**
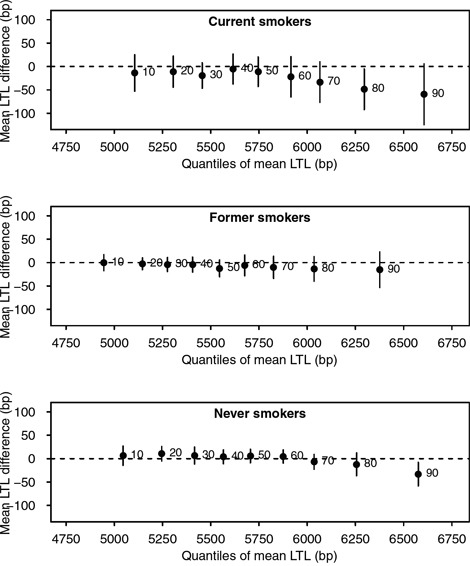
Associations between log-transformed cotinine levels and quantiles of mean LTL, stratified by self-reported smoking status All models were adjusted by age, sex, race/ethnicity, PIR, education, BMI, vigorous physical activity, and alcohol intake. The x-axes represent mean LTL value, and y-axes represent mean LTL difference for every log unit increase in cotinine levels. Black points indicate estimates for each decile, represented by adjacent numbers. Error bars represent 95% confidence intervals from bootstrap resampling.

We performed quantile regression analysis to further investigate the association between cotinine levels and LTL by self-reported smoking status. Overall, an inverse trend was observed between log-transformed cotinine and LTL among higher quantiles of telomere length across all groups, which suggested non-linear associations. In particular, shorter LTL in the 90^th^ quantile was shown with increasing log-transformed cotinine levels among never smokers, with a 33 bp decrease (95% CI: −61 to −4). Similarly, an inverse association was shown between log cotinine and LTL in the 80^th^ and 90^th^ quantiles (Figure 1). No associations were observed among former smokers. When we further adjusted the models with pack-year of cigarette for current smokers, the observed association among current smokers was no present (results not shown).

**Figure 2:**
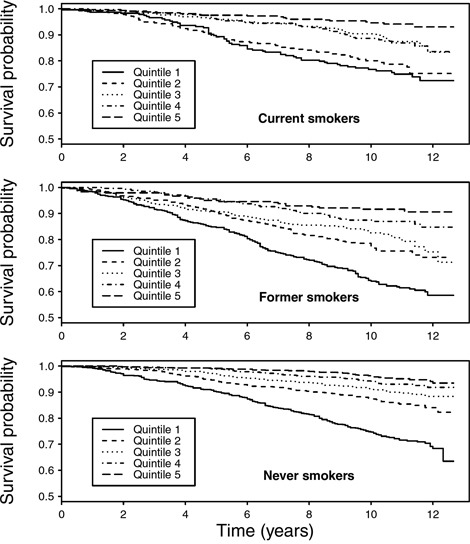
Kaplan-Meier curves for overall survival by quintiles of mean LTL (T/S ratio), stratified by self-reported smoking status at baseline

**Figure 3:**
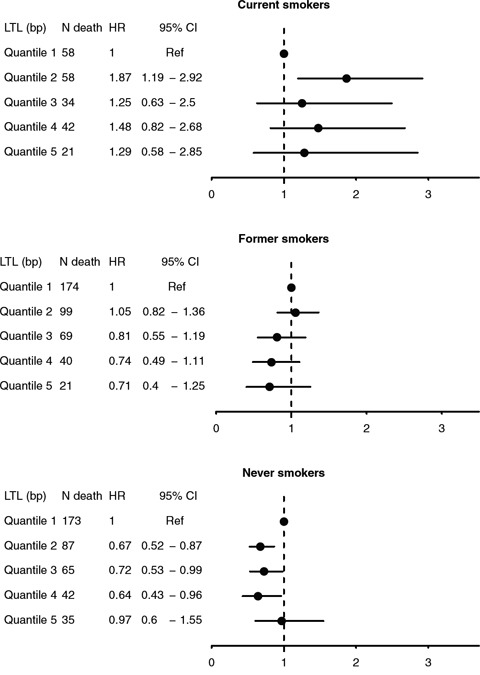
Hazard ratios (HRs) and 95% confidence intervals for all-cause mortality by quintiles of mean LTL (T/S ratio), stratified by self-reported smoking status at baseline

We assessed the interplay between smoking status and LTL in relation to all-cause mortality. As shown in Kaplan-Meier curves (Figure 2), lower overall survival was observed with shorter telomere length for all smoking categories, particularly among never smokers (P_log-rank_ <0.05). In multivariable Cox regression, no associations were found LTL and risk of early death overall was found. In stratification analyses, weak inverse trends between LTL quintiles and risk of early death were observed among self-reported former and never smokers (Figure 3). A statistically significant lower risk of death was observedfor the 2^nd^, 3^rd^ and 4^th^ quintiles of LTL among never smokers (e.g. HR: 0.64, 95% CI: 0.43–0.96 for the 4^th^ quintile compared to the 1st). In contrast, a higher risk was observed among current smokers in the 2^nd^ quintile of LTL (HR: 1.87, 1.19–2.92). Results were similar with cotinine-confirmed smoking status (results not shown).

**Figure 4:**
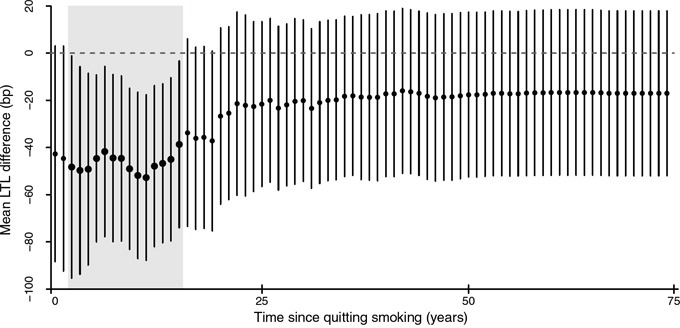
The dynamic of smoking cessation and telomere length For any given *t* representing time since quitting smoking, mean LTL was compared between smokers, comprising current smokers and former smokers who quitted smoking up to *t* years ago, and non-smokers, which included never smokers and former smokers who quitted smoking more than *t* years ago. Points and lines indicate estimates of mean LTL difference in base pair between smokers and non-smokers for each *t* and their 95% confidence intervals, respectively. Bold points and shaded areas represent statistically significant differences.

To further investigate the role of smoking cessation duration, we compared LTL for each *t* indicating time lag since abstaining or quitting smoking, between recoded smokers, i.e. current smokers and former smokers quitting <*t* years, and non-smokers which comprised never smokers and former smokers quitting ≥*t* years. As shown in Figure 4, we observed shorter LTL among recoded smokers compared to non-smokers with 3 to 16 years of time since smoking cessation. No difference between the two recoded groups was observed beyond this time interval. For all-cause mortality, a similar analysis showed consistently higher risk of death among recoded smokers as compared to non-smokers without any marked difference between time intervals since abstaining from smoking (results not shown).

## DISCUSSION

Our findings strengthen evidence for an association between shorter telomere length with increasing smoking intensity. Increased cotinine levels corresponded to shorter telomeres among never smokers in higher quantiles of LTL, indicating non-linear associations and the role of SHS exposure. Among former smokers, a borderline inverse association was seen between telomere length and time after quitting smoking. The association between smoking cessation and LTL was further suggested to be strongest within 3 to 16 years of abstaining smoking. When assessing mortality, we found effect modification by smoking status in association between LTL and all-cause death.

To date, evidence regarding the association between SHS exposure or smoking cessation and telomere length remains scarce. In our study, mean LTL was longer with exposure to SHS compared to a lack of it, although no marked difference was found for either home or workplace SHS exposure. However, we observed shorter telomeres following increased cotinine levels among higher quantiles of LTL, indicating non-linear associations. Non-linear patterns have been observed between other biological measures and health outcomes, for instance the J-shape curve observed between alcohol intake [18] or vitamin D [19] and mortality. As with these two lifestyle-related exposures, understanding of such associations is necessary for targeted interventions.

We found no difference in LTL between self-reported current, former and never smokers. This inconsistency has been previously reported (Supplementary Table S3), and several possible explanations are as follows. First, smoking status is mostly assessed by self-reported history which is prone to recall bias among other biases [20]. We addressed this by using cotinine-confirmed categories and observed slightly stronger associations overall between smoking exposures and LTL. Secondly, non-linear associations between smoking exposures and LTL may occur as previously mentioned. Finally, although telomere shortening is regarded as an indicator of ageing, the clinical and public health relevance of LTL as a biomarker of longevity is not fully understood [21]. In the NHANES population, this was shown by a lack of clear trends between LTL and mortality overall. Furthermore, our study showed effect modification of this association by smoking status, implying differential biological or socioeconomic pathways involved, which require mechanistic investigation.

The present study implies that smoking cessation may be associated with less telomere attrition. A similarly positive association between duration of smoking cessation and telomere length has been reported in a study among older adults (50–75 years) in Germany [22]. Correspondingly, favourable age-related biological and health outcomes have been reported to follow smoking cessation, including decreased levels of inflammatory biomarkers [23], and reversal of lung and heart function, [24, 25] which may be accounted for by epigenetic changes [26]. Telomere elongation may also indicate reactivation of telomerase pathways as reported in some malignancies [27]. However, changes associated with malignant processes tend to be organ- or tissue-specific rather than systemic, and we found similar results after excluding cancer diagnosis. Alternatively, our results may represent certain smoking behaviours. For instance, relapse rate is highest (~60%) in the first three years of smoking cessation, [28] which may explain a lack of meaningful results for short-term cessation. Despite adjustment for age and lifestyle, a lack of association with longer periods of smoking cessation may have been unclear due to cumulative DNA-damaging effects from other exposures with later age [27]. Confirmation of any causal effect of smoking cessation on telomere length thus requires longitudinal data collection, which was beyond the scope of this study.

The strength of this study lies on the nationally representative sample included in the NHANES surveys. Our analysis included both self-reported history and objective assessment of nicotine exposure by measuring serum cotinine levels. One of the limitations of this study is that information was based on cross-sectional surveys. Whilst we were able to evaluate duration of smoking cessation, our findings only implied association rather than causation. Misclassification may have occurred since information on causes of death was collected by means of probabilistic matching [29]. However, such bias would be non-differential. The low number of cases also hampered analyses on specific causes of death. Although NHANES was designed to represent the general population of the US, those with higher exposure of active or second-hand smoking have increased mortality risk and are therefore more likely to die before being included in the surveys compared to less exposed or unexposed individuals. This may have led to an underestimation of associations observed in this study. Spurious correlations may also be of concern due to the small number of events which hampered statistical power in our subgroup analyses of mortality, prompting the need to confirm these findings in studies with longer follow-up period. Another limitation is that telomere length may be altered in carcinogenesis [30]. Although findings were similar when analyses were restricted to those without history of cancer or COPD, residual confounding may have occurred due to lack of information on cancer incidence and definitive diagnoses of COPD.

In summary, our findings corroborate associations between smoking exposures, biological ageing and mortality, and indicate the impact of second-hand smoke exposure and smoking cessation on ageing. Further investigations on biological and socioeconomic pathways underlying these findings may identify targets for intervention, and inform future clinical and public health policies aimed to reduce the burden caused by the smoking.

## MATERIALS AND METHODS

### Study population

The National Health and Nutrition Examination Survey (NHANES) is a cross-sectional health survey conducted by the National Center for Health Statistics (NCHS) in representative samples of the non-institutionalized U.S. population [31]. Participants were selected through multistage stratified, clustered probability sampling. The survey included an interview conducted at home and an extensive physical examination, which included a blood sample taken in a mobile examination centre (MEC). This study was based on the continuous NHANES 1999–2002, which included 21,004 participants, among whom 10,291 were 20 years old and above. From this population, we selected 10,262 adults who had complete data on self-reported smoking status. We subsequently excluded pregnant women (N=603) and those without available data on LTL measurements (N=2,313) or other covariates: poverty-to-income ratio, education level, body mass index (BMI), vigorous physical activity, and alcohol intake (N=890). Characteristics of study participants (age, sex, race/ethnicity) did not differ when missing values were excluded. We assumed missing values to have occurred completely at random and complete cases (N=6,456) were used in the analysis.

### Telomere length assay

The telomere length assay was performed in the laboratory of Dr. Elizabeth Blackburn at the University of California, San Francisco, using the quantitative polymerase chain reaction (qPCR) method to measure telomere length relative to standard reference DNA (T/S ratio), as described in detail elsewhere [16, 32]. Each sample was assayed three times on three different days. The samples were assayed on duplicate wells, resulting in six data points. Sample plates were assayed in groups of three plates, and no two plates were grouped together more than once. Each assay plate contained 96 control wells with eight control DNA samples. Assay runs with eight or more invalid control wells were excluded from further analysis (<1% of runs). Control DNA values were used to normalize between-run variability to control for potential batch effects. Runs with more than four control DNA values falling outside 2.5 standard deviations from the mean for all assay runs were excluded from further analysis (<6% of runs). For each sample, any potential outliers were identified and excluded from the calculations (<2% of samples). The mean and standard deviation of the T/S ratio were then calculated normally. The interassay coefficient of variation was 6.5%. T/S ratio was converted into base pairs (bp) using the following formula: (3,274 + 2,413 * (T/S)) [33]. The conversion from T/S ratio to bp was calculated based on comparison of telomeric restriction fragment (TRF) length from Southern blot analysis and T/S ratios using DNA samples from the human diploid fibroblast cell line IMR90 at different population doublings.

### Mortality follow-up

Information on dates of death was obtained from data linkage of the NHANES dataset with the National Death Index (NDI). This linkage was performed by the NCHS through probabilistic matching with social security number, birth date, occupation, and other personal data, and confirmation with death certificate when possible [29]. Follow-up time was calculated from interview date/examination date until date of death or end of study (31 December 2011), whichever came first.

### Assessment of smoking exposures

Participants were asked whether they had smoked at least 100 cigarettes in their entire life, and those who responded positively were asked whether they now smoke cigarettes every day, some days, or not at all. We defined current smokers as those who had smoked at least 100 cigarettes during their lifetime and, at the time of the interview, reported smoking either every day or some days. Former smokers were those who reported smoking at least 100 cigarettes during their lifetime but currently did not smoke. Never smokers were those who had not smoked 100 cigarettes during their lifetime. We also identified SHS exposure at home and at workplace, defined as never smokers who answered ‘yes’ to the question “Does anyone who lives here smoke” or admitted that they could smell other people's smoking at workplace, respectively. In addition to self-reported history, we additionally used measurement of serum cotinine, a major metabolite of nicotine [34] to construct a secondary smoking status. Serum cotinine (ng/mL) was measured by an isotope dilution-high performance liquid chromatography/atmospheric pressure chemical ionization tandem mass spectrometry [35]. For the cotinine-confirmed smoking status, those with serum cotinine 10 ng/mL or above were considered current smokers regardless self-reported history, and never smokers with cotinine levels of 0.05 ng/mL were regarded as being exposed to SHS [34, 36]. We further characterised former smokers based on the length of time since they quit smoking, years since started smoking and total cigarette smoked in a day. Former smokers were grouped by years since they quitted smoking and total cigarette smoked in a day when quitting. For both current and former smokers, pack-year of cigarette was calculated by dividing total cigarette smoked per day by total cigarette per pack, i.e. 20, and multiplying the result by the duration of smoking in years. We did not assess the use other types of tobacco such as cigars and snuff given the small number of participants using these products.

### Other covariates

Race/ethnicity was categorised into Non-Hispanic white, Non-Hispanic black, Mexican-American, and others. We classified educational attainment as less than high school, high school equivalent, and higher than high school. SES was estimated with poverty-to-income ratio (PIR), a ratio of total family income to the official poverty threshold at the family level. A PIR <1 indicated that income was less than the level of poverty. We categorised PIR into <1, 1–2, 2–3, and ≥3, indicating lowest to highest SES [37]. A self-reported history of cancer diagnosis was based on a positive response to the question “Have you ever been told by a doctor or other health care professional that you had cancer or a malignancy of any kind”. Similarly, chronic obstructive pulmonary disease (COPD) was defined as self-reported history of chronic bronchitis or emphysema. Weight was measured with an electronic weight scale in pounds and automatically converted to kilograms. Participants only wore underwear, disposable paper gowns and foam rubber slippers. Standing height was measured with a fixed stadiometer to the nearest 1 mm. Body mass index (BMI) was calculated from weight and height and classified into less than 18.5, 18.5–25, 25–30, and 30 kg/m^2^ or greater [38]. Vigorous physical activity was described as reporting three or more physical activities of at least six metabolic equivalents (METs) per week, each with a duration of at least 10 minutes [39]. Frequency of alcohol consumption was collected during interview and categorised into never (had fewer than 12 alcohol drinks throughout their life), up to once a week, 2–3 times a week, and 4 times a week or more.

### Statistical analysis

We estimated proportions of participant characteristics and corresponding mean LTL with the NHANES 2001–2002 sampling weights for genetic data subsample. Mean LTL (T/S ratio) in base pair was assessed as the outcome. First, we determined the associations between self reported smoking status and LTL using an age-, sex- and race/ethnicity-adjusted linear regression model. In the second model, we additionally adjusted for categories of PIR and education level given the impact of socioeconomic status on population health [40, 41]. Adjustments for BMI, vigorous physical activity and alcohol consumption were further performed in the fully adjusted model, which was used for all subsequent analyses. A similar analysis was performed for cotinine-confirmed smoking status.

We assessed the quantity and intensity of smoking, expressed as pack-year of cigarettes, and smoking duration for both current and former smokers, and duration since quitting smoking for former smokers only. SHS exposure at home and workplace was assessed for never smokers, and log-transformed levels of serum cotinine were in each group. Since altered telomere length has been observed with some malignancies and COPD, [30, 42] and given common pathways linked to smoking and these diseases such as inflammation, [43–46] a sensitivity analysis was performed by excluding participants reporting any history of cancer diagnosis or COPD. The NHANES data was prepared with Statistical Analysis Software (SAS) release 9.4 (SAS Institute, Cary, NC) and survey-weighted analyses were performed with the survey package of R version 3.1.2 (R Foundation for Statistical Computing, Vienna, Austria).

To further characterise the relationship between smoking, SHS exposure and telomere length, we performed quantile regression assessing difference in deciles of LTL with increasing log-transformed cotinine. This analysis predicted changes in a given quantile, e.g. median (0.5) of outcome variables instead of their mean, allowing identification of non-linear associations. [47] All models were adjusted for age, sex, race/ethnicity, PIR, education, BMI, vigorous physical activity and alcohol consumption. Analyses were stratified by smoking status (current, former and never smokers). For current smokers, we performed a sensitivity analysis by controlling for pack-year of smoking since smoking intensity may affect both cotinine levels [35] and telomere length. [16] The R quantreg package incorporating NHANES sampling weight replicates were used to obtain quantile regression estimates. Ninety-five percent confidence intervals were obtained using 100 bootstrap re-samples.

To gain further insight into associations between smoking exposures, telomere length and mortality, we displayed survival probability by LTL quintiles and smoking status using Kaplan-Meier curves. We further estimated hazard ratios (HRs) and 95% confidence intervals (CIs) of all-cause mortality by continuous values and quintiles of LTL for self-reported current, former, and never smokers. All models were adjusted for age, sex, race/ethnicity, PIR, education, BMI, vigorous physical activity and alcohol consumption. Analyses were repeated with cotinine-confirmed smoking status, and additional adjustment for pack-year of smoking was performed for current smokers. Due to the insufficient number of cases, we were unable to perform analyses by specific causes of death or characteristics of smoking exposures.

Finally, we sought to gain insight into the effect of smoking cessation dynamics on telomere length and risk of mortality, using duration since abstaining or quitting smoking as a proxy and a recoded binary variable for smoking status (smokers and non smokers). This approach has been previously used to investigate patterns of DNA methylation following smoking cessation. [26] For each value of *t*, reflecting the time since smoking cessation, those assigned as ‘smokers’ included current smokers and former smokers who quitted smoking <*t* years ago, and ‘non-smokers’ comprised never smokers and former smokers who quitted ≥*t* years ago. We employed survey-weighted multivariable linear regression to compare LTL in base pairs between recoded smokers and non-smokers for each *t*. Therefore, for t=0, we assessed LTL among all current smokers with former and never smokers combined as the referent group, and for the highest *t*, we compared LTL among ever smokers against never smokers. Similarly, risk of dying from all causes was compared between the two recoded groups for each *t*. All models were adjusted for age, sex, race/ethnicity, PIR, education, BMI, vigorous physical activity and alcohol consumption.

## SUPPLEMENTARY FIGURE AND TABLES




