# Supplementary material for: Smoking, second-hand smoke exposure and smoking cessation in relation to leukocyte telomere length and mortality
Source: Oncotarget. 2016 Aug 4;7(37):60419–31. doi: 10.18632/oncotarget.11051 (PMC5312393; doi:10.18632/oncotarget.11051)
Supplement: Supplementary file 1 [file oncotarget-07-60419-s001.pdf]

Smoking, second-hand smoke exposure and smoking cessation in relation to leukocyte telomere length and mortality

SUPPLEMENTARY FIGURE AND TABLES

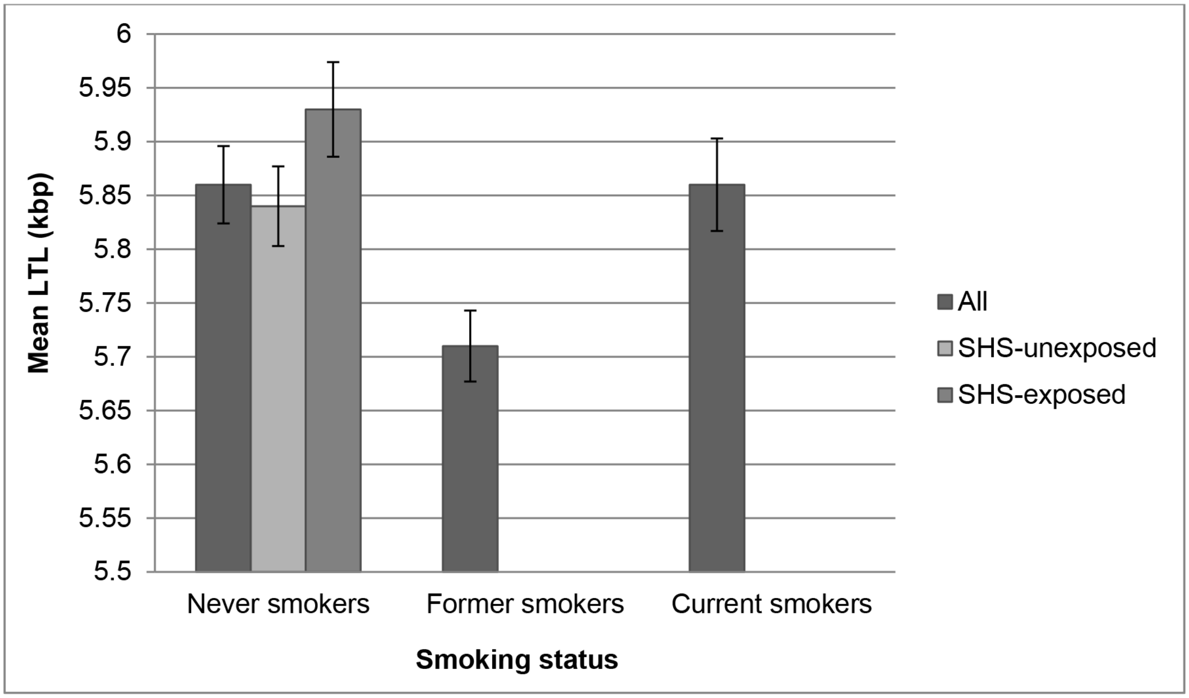

Supplementary Figure S1: Weighted mean telomere length in kilo base pairs (kbp) among current, former and never smokers, overall. Never smokers were stratified by any self-reported SHS exposure (either at home or workplace).

Supplementary Table S1: Univariable associations between covariates and mean LTL in base pairs

	Mean LTL difference (bp)	95% CI
**Age, years**	-14	-16, -13
**Sex**		
Male	Reference	
Female	11	-32, 55
**Race/ethnicity**		
Non Hispanic white	Reference	
Non Hispanic black	160	83, 236
Mexican American	24	-95, 144
Other	104	-55, 265
**PIR**		
**Education**		
Less than high school	Reference	
High school	80	20, 140
Higher education	141	81, 201
**Vigorously active**		
No	Reference	
Yes	176	116, 234
**Body mass index (BMI), kg/m^2**	-9	-12, -5
**Alcohol consumption**		
Never	Reference	
Up to once a week	-110	-247, 27
2–3 times per week	-7	-186, 171
4 times per week or more	-139	-299, 19

Supplementary Table S2: The association between smoking status and mean LTL in base pairs

	Model 1[1]		Model 2[2]		Model 3[3]	
	Mean LTL difference (bp)	95% CI	Mean LTL difference (bp)	95% CI	Mean LTL difference (bp)	95% CI
**Self-reported history**						
**Current smokers**						
vs former smokers	-37	-87 to 12	-25	-73 to 22	-35	-86 to 15
vs never smokers	-50	-105 to 5	-36	-88 to 17	-35	-85 to 15
**Former smokers**						
vs never smokers	-12	-51 to 27	-10	-47 to 27	0.2	-35 to 36
**Cotinine-confirmed**						
**Current smokers**						
vs former smokers	-42	-90 to 5	-32	-78,13	-42	-90 to 6
vs never smokers	-55	-102 to -8	-43	-86 to 1	-43	-85 to -1
**Former smokers**						
vs never smokers	-14	-59 to 32	-12	-54 to 31	-1	-41 to 38

[1]Adjusted for age (continuous), sex, and race/ethnicity.
[2]Adjusted for age (continuous), sex, race/ethnicity, PIR, and education.
[3]Adjusted for age (continuous), sex, race/ethnicity, PIR, education, BMI (continuous), vigorous physical activity, and alcohol consumption.

Supplementary Table S3: Overview of previous studies on smoking and telomere length

See Supplementary File 1
