# Supplementary material for: Smoking, second-hand smoke exposure and smoking cessation in relation to leukocyte telomere length and mortality
Source: Oncotarget. 2016 Aug 4;7(37):60419–31. doi: 10.18632/oncotarget.11051 (PMC5312393; doi:10.18632/oncotarget.11051)
Supplement: Supplementary file 2 [file oncotarget-07-60419-s002.docx]

**Table S3**. Overview of previous studies on smoking and telomere length

| **Study** | **Population** | **Smoking assessment** | **Telomere measure & source** | **Results** | **Notes** |
| --- | --- | --- | --- | --- | --- |
| Latifovic, 2016 | Cross-sectional study of healthy volunteers, age 20-50, from Ontario and Canada, 477 individuals | Self-administered research questionnaire | rLTL, multiplex quantitative real-time PCR, leukocyte | Daily smokers and those in the middle and lower tertile of pack-years smoking had shorter rLTL than never daily smokers (p = 0.02) | Multivariable models were adjusted for age, sex, and lipid ratio |
| Verde, 2015 | Sample of healthy smokers, Caucasian Spanish descent, age 25-65, total of 147 volunteers | Self-reported questionnaire; Fagerstrom Test for Nicotine Dependence to asses nicotine dependence | rLTL (36B4 gene as control), quantitative real-time PCR, leukocyte | Association between greater smoking intensity (PYS) and shorter telomere length (OR = 2.92, 95% CI = 1.09 – 7.81) | Nicotine metabolism ratio (NMR), hypothesized to be measure of toxin exposure, displayed no association with shorter telomere length |
| Huzen, 2014 | Prevention of Renal and Vascular End-stage Disease (PREVEND) study, Netherlands, men and women aged 28-75 years at baseline | Self reported at baseline: Current, previous and non smoking (definition N/A) | Relative telomere length (rLTL), qPCR, leukocyte  Annual telomere attrition from 2 and 3 repeated measurements | Cross-sectional samples (univariable model): β for rLTL = -2.20 (95%CI: -3.37 to –1.03) for each log increase in no. of cigarettes/day  Longitudinal sample (multivariable model): β for LTL attrition = 0.67(0.37-0.98) for smokers vs nonsmokers | Multivariable models were adjusted for age, sex, baseline rLTL, waist-to-hip ratio, HDL and glucose levels |
| Rode, 2014 | Copenhagen General Population Study, Denmark, men and women aged 20-100, 55,568 individuals of which 3,430 died during 10 years of follow-up | Self-reported questionnaire reviewed together with an examiner: divided into never smokers and ever smokers | rLTL, multiplex quantitative real-time PCR, leukocyte | High tobacco consumption and short telomeres were associated with all-cause mortality. However, while high tobacco consumption was causally associated with all-cause mortality, there was no clear causal association between tobacco consumption and short telomeres. | Sample population was also classified according to CHRNA3 genotype as an instrument in a Mendelian randomization design |
| Weischer 2014 | Copenhagen City Heart Study, Denmark, men and women, aged 20 -100, 4,576 participants in a longitudinal study in 1991-94 and 2001-03 | Self-administered questionnaires with an examiner: classification between current smoking (yes/no) | rLTL, multiplex quantitative real-time PCR, leukocyte | Cross-sectional analysis in 1991-1994 and 2001-2003: conflicting results with short telomere length associated with current smoking (p = 0.008) in the former and not in the latter  Longitudinal analysis: tobacco consumption was not associated with change in telomere length | Multivariable models were adjusted for age, sex, baseline rLTL, BMI, heavy alcohol intake and physical activity |
| Müezzinler, 2014 | Epidemiological Study on the Chances of Prevention, Early Recognition, and Optimised Treatment of Chronic Diseases in the Older Population (ESTHER), Germany, men and women, aged 50-75, 3,600 participants with available baseline LTL | Standardized detailed questionnaire during a general health check-up: categorized between current smokers, formers smokers and never smokers | rLTL, multiplex quantitative real-time PCR, leukocyte; validation with terminal restriction fragment Southern Blot Analysis | Cross-sectional multivariate analysis: current smokers had on average 73bp shorter LTL compared to never smokers (p < 0.0001); former smokers also tended to have shorter LTL compared to never smokers but this was not statistically significant  Longitudinal sample: ever smokers showed slower LTL attrition rates than never smokers with current smokers showing the slowest rate | Depending on the model used, analysis was increasingly adjusted for sex, age, batch effect (Model 1), BMI, alcohol consumption, years of education and physical activity (Model 2), hypertension, hypercholesterolemia, diabetes, history of myocardial infarction, cancer, stroke, and depression (Model 3). |
| Needham, 2013 | The National Health and Nutrition Examination Survey (NHANES), United States of America, nationally representative of 21,004 individuals aged 2 months and older | Interview | Relative telomere length (rLTL), RT-PCR, leukocyte; human bet-globin as input DNA control | Cross-sectional samples (regression model):  60 pack-years of smoking or more had significantly shorter telomeres than never-smokers  (b = -0.039, p < 0.05) | Socio-economic status (SES; education and income) was hypothesized to be positively associated with LTL with health behaviours such as smoking among others as partial mediators between SES and LTL |
| Strandberg, 2011 | The Helsinki Businessmen Study, Finland, 3,490 healthy Finnish men aged 30-45 years at baseline | Self-reported through mailed questionnaires between 1985 and 2010 | TeloTAGGG Telomere length assay kit (Roche Molecular Biochemicals), Southern blotting measuring signal intensity below 5kb | Age-adjusted TL was significantly longer among life-long never-smokers vs past or present smokers  (p = 0.0004); significant difference in proportion of short telomeres (p = 0.05) | Combined with BMI, smoking was age-independently and in a graded manner associated with shorted LTL in old age |
